# Sparse Matrix for ECG Identification with Two-Lead Features

**DOI:** 10.1155/2015/656807

**Published:** 2015-04-16

**Authors:** Kuo-Kun Tseng, Jiao Luo, Robert Hegarty, Wenmin Wang, Dong Haiting

**Affiliations:** ^1^Shenzhen Key Laboratory of Internet Information Collaboration, Shenzhen Graduate School, Harbin Institute of Technology, Shenzhen, Guangdong 518052, China; ^2^School of Computing and Mathematical Sciences, Liverpool John Moores University, Liverpool L3 3AF, UK; ^3^School of Electronic and Communication, Shenzhen Graduate School, Peking University, Shenzhen, Guangdong 518052, China

## Abstract

Electrocardiograph (ECG) human identification has the potential to improve biometric security. However, improvements in ECG identification and feature extraction are required. Previous work has focused on single lead ECG signals. Our work proposes a new algorithm for human identification by mapping two-lead ECG signals onto a two-dimensional matrix then employing a sparse matrix method to process the matrix. And that is the first application of sparse matrix techniques for ECG identification. Moreover, the results of our experiments demonstrate the benefits of our approach over existing methods.

## 1. Introduction

Electrocardiogram (ECG) has become a popular tool in analyzing heart disease with the use of telemedicine and home care techniques [1, 2]. However, ECG not only is useful as a diagnostic tool but also has been applied on information watermarking [3–5], data compression [3, 6], and human identification [7–15]. ECG techniques have the potential to play a role in biometric identification.

Existing biometric identification techniques have focused on the use of fingerprints, facial geometry, and voice analysis. We may be able to apply ECG techniques to protect health care systems from data leakage.

In this work, we propose a new algorithm using two leads of ECG signals for human identification. This algorithm uses the sparse matrix for dimensionality reduction that mapped two-lead data into one coordinate. We take advantage of the sparse matrix for identification. Our algorithm is sparse matrix correlation coefficient (SMCC).

Through the experiment, we demonstrate that our approach is more accurate for human identification and verification than existing techniques. In a summary, compared to the previous ECG identification, our approach has the following advantages.

(1) Using sparse matrix to store data that contains a large number of zero-valued elements can both save a significant amount of memory and speed up the processing of that data.

(2) The algorithm performs rapidly with lower computational complexity than the PCA method to process two-lead signals.

The remainder of this paper is organized as follows: Section 2 contains an overview of related work in ECG identification; Section 3 introduces the proposed ECG identification algorithm; the experimental results are presented in Section 4; finally, some concluding remarks are stated in Section 5.

## 2. Related Work

For ECG identification, research has focused on areas such as signal preprocessing, feature extraction, data classification, data reduction, and intelligence optimization.

Based on our survey, the ECG feature extraction algorithms can be classified into two categories: transform-based [9, 13, 15] and waveform-based [7, 8, 11, 12]. The transform-based algorithms consist of transforms in wavelet [15] and frequency domain. Since the wavelet transform contains information in both the time and frequency domains, it is more popular than the frequency based techniques which include Fourier transform [13] and discrete cosine transform (DCT). Waveform-based method measures the distance and amplitude difference between wave peaks and valleys. These attributes represent certain characteristics of the signal, such as in [8]; morphological characteristics are first extracted through the wavelet transform.

But some approaches are hybrid, for example [8], using morphological characteristics which are extracted through the wavelet transform. The feature extraction of our approach could be a hybrid approach as well; it transfers two waveform signals into a two-dimensional space then measures their similarity in the two-dimensional space.

Most approaches to ECG identification only use one-lead signal [7, 10, 14]. Lead systems allow you to look at the heart from different angles. Each different angle is called a lead. The different leads can be compared to radiographs taken from different angles. So we can use more than one feature to classify discrimination. In many characteristics classification, there are many kinds of features.

Two-dimensional processing of ECG data has been applied in compression and diagnostic areas. The authors of [16] proposed diagnosis of acute myocardial infarction using two-dimensional echocardiography. The authors of [17, 18] implemented ECG data compression on two-dimensional data. These approaches have however to our knowledge not been applied to ECG identification.

In a summary, most of the work on ECG biometrics made use of only one lead and ignored the other leads that may contain additional information. The ECG signals from the two leads are essentially two observations of the same physiological activity from two different perspectives. Thus, we proposed a new two-lead algorithm for ECG identification.

Data computation is another area that we reviewed when considering existing approaches to ECG identification. A common approach is correlation of coefficients for measurements of feature distance, such as the wavelet distances that have been used in matching acquired ECG signals for identification [9, 15]. The work [19] applied the feature set evaluation (FSE) with* k*-nearest neighbor (*k*-NN) algorithm to improve low recognition rates and used the eigen-space method to reduce data dimensions; however, this approach is both complicated and time consuming. By using typical neural classifier, the research [11, 20] is applying the neural network in ECG identification.

Further, one popular approach is PCA [21] which is an analogue of the principal axes theorem in mechanics, and it was later independently developed by [22]. A recent application of PCA in ECG signal processing is useful feature reduction of various ECG properties [1, 2, 8, 23].

In this work, we adapt spare matrix [24] for ECG identification, and it was invented as early as a century ago; CF Gauss, CGJ Jacobi, and others have studied the use of matrix sparse in some ways. Linear programming and numerical solution of boundary value problems had been apply for sparse problems in 1950s. DM Young and RS Varga on iterative research process can also be seen as the result of high-level sparse problem. But modern sparse matrix technology is mainly developed since the 1960s, and in the early and mid-60's some researchers studied the direct method as a starting point. Sparse matrix has penetrated into many areas of research. For example, in structural analysis, network theory, power distribution systems, chemical engineering, photography, surveying and mapping, and other aspects of management science studies have appeared until hundreds of thousands of rank-order sparse matrix.

But according to our survey, we did not find any one to transform ECG signal into two-dimensional space and fuse with sparse matrix. In this research, we also found that they work well for the similarity measurement in ECG identification.

## 3. Algorithm

**Figure 1:**
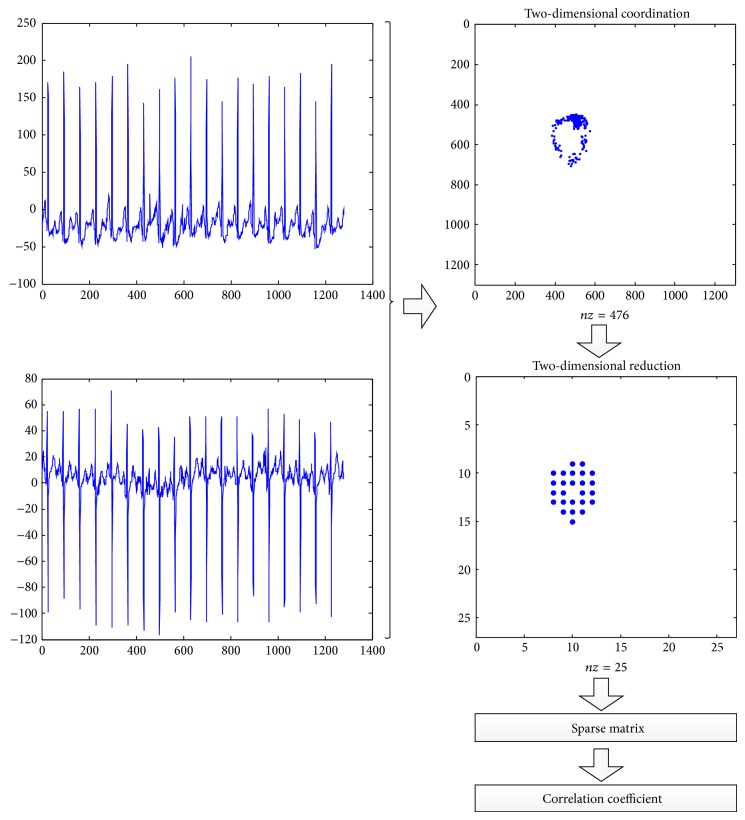
Procedure of ECG identification system with sparse matrix.

In this work, we target the two-lead ECG signal to be transformed into two-dimensional coordinates and perform the identification using sparse matrix.  Figure 1 shows the flow of utilizing sparse matrix in ECG human identification system, which consists of three steps. First, we map the ECG two-lead signals into two-dimensional coordination that forms a matrix. Then, we reduce dimensions of the matrix using a special mask, the size of which depends on how many dimensions we want to reduce. We then transfer the matrix into a sparse matrix so that it can be stored and addressed easily. The sparse matrix is regarded as the fusion features of ECG two-lead signals. Finally, the feature data for various individuals are used to train the sparse matrix classifier.  Figure 1 is the detailed formula for the procedure.

*(1) Obtaining Two-Lead ECG Signals*. Consider two-lead ECG signals, respectively, as

(1) $$\begin{array}{c} S^{\left(1\right)}=\left\{x_{1}^{\left(1\right)},x_{2}^{\left(1\right)},\ldots ,x_{i}^{\left(1\right)},\ldots ,x_{n}^{\left(1\right)}\right\},\\ {\;S}^{\left(2\right)}=\left\{x_{1}^{\left(2\right)},x_{2}^{\left(2\right)},\ldots ,x_{i}^{\left(2\right)},\ldots ,x_{n}^{\left(2\right)}\right\}, \end{array}$$

where real-valued *x*_*i*_^(*j*)^  (*j* = 1,2) corresponds to the *i*th of the *j*th ECG leads signal.

*(2) Transforming ECG Signals into Matrix*. Then, we initialize a matrix *M* to zero, and then compare each pair of consecutive input signals at time  *t*, and set

(2) $$\begin{array}{c} M\left[x_{t}^{\left(1\right)},x_{t}^{\left(2\right)}\right]=1. \end{array}$$

In this procedure, we converted each lead signals of ECG to a matrix *M* whose size is *N* × *N*. Here, *N* is the scopes of signal values.

*(3) Reduced Matrix*. Next, we defined a mask matrix *M* ^∗^^*m*×*m*^,  (*m* < *N*),

(3) $$\begin{array}{c} M^{*}=\left[\begin{array}{ccc} 0 & \cdots & 0\\ \vdots & \ddots & \vdots \\ 0 & \cdots & 0 \end{array}\right], \end{array}$$

where *m* means the dimensions that we want to reduce. For *x* coordination *k*_*x*_ = 1,1 + *m*, 1 + 2*m*,…, *N* − *m* + 1, and *y* coordination *k*_*y*_ = 1,1 + *m*, 1 + 2*m*,…, *N* − *m* + 1, we follow the rule:

(4) MRkx+m−1m,ky+m−1m =0,Mkx:kx+m−1,ky:ky+m−1+M∗≤0,1,Mkx:kx+m−1,ky:ky+m−1+M∗>0.  

*(4) Storing as a Sparse Matrix*. And then we store the matrix as sparse matrix for the following processing.

**Figure 2:**
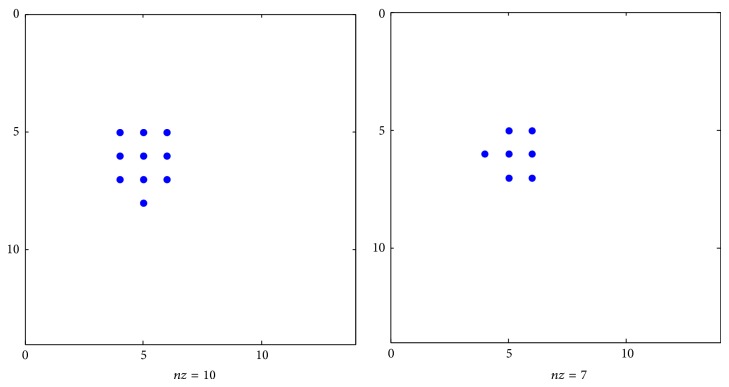
Extracted ECG two-lead signals data.

Here, we use the coordinate format (COO) to store the spare matrix; that is, we just store three parameters, row, column, and value. As most elements of the matrix are zero and we set the corresponding point as “1,” we can just only use (row, column) sparse matrix format to express the full matrix of extracted ECG two-lead signals data. For an example, after processing, we get the ECG data that we extracted from the two-lead signals as Figure 2 shows, where  *nz* means number of elements of this sparse matrix.

We can express the sparse matrices as

(5) val=5,4,6,4,7,4,5,5,6,5,7,5,hhh8,5,5,6,6,6,7,6,val=6,4,5,5,6,5,7,5,5,6,6,6,7,6.

The relative sparse matrix val is considered as the features of ECG two-lead signals and will be the input to the correlation coefficient classifier for training purposes and individual identification.

*(5) Computing Correlation Coefficient*. *R* = corrcoef(*X*) returns a matrix *R* of correlation coefficients calculated from an input matrix *X* whose rows are observations and whose columns are variables. The matrix *R* = corrcoef(*X*) is related to the covariance matrix *C* = cov(*X*) by

(6) $$\begin{array}{c} R\left(i,j\right)=\frac{C\left(i,j\right)}{\sqrt{C\left(i,i\right)C\left(j,j\right)}}. \end{array}$$

cov removes the mean from each column before calculating the result.

The covariance function is defined as

(7) $$\begin{array}{c} \mathrm{cov}\left(\vec{x_{1}},\vec{x_{2}}\right)=E\left[\left(\vec{x_{1}}-\mu_{1}\right)\left(\vec{x_{2}}-\mu_{2}\right)\right], \end{array}$$

where $\vec{x_{i}}$ is a vector, *E* is the mathematical expectation, and $\mu_{i}=E\vec{x_{i}}$.

For an ECG data signal  *S* = {*s*_1_, *s*_2_,…, *s*_*n*_}, which is from one unidentified individual. After transferring *S* into a sparse matrix *ℳ* ^~^, we calculated the correlation coefficient of *ℳ* ^~^, *ℳ*_*i*_, respectively, where *ℳ*_*i*_ is the template sparse matrix that came from a special individual, and we get *r*_*i*_ = corrcoef (*ℳ* ^~^, *ℳ*_*i*_). If we have *n* individuals, then we have an output result$\;\vec{R}=\{r_{1},r_{2},r_{3},\ldots ,r_{n}\}$. In sparse matrix correlation coefficient classification, we define *ε*_*i*_ as a threshold of *i*th individual. If *r*_*i*_ < *ε*_*i*_,  (*i* ∈ 1,2,…, *n*), it means we can classify *S* to this corresponding target individual type *i*; that is, *S* can be classified into the right type only if it belongs to a unique right type.

*(6) Setting Threshold for Identification*. To verify how efficient this algorithm is in human identification by ECG two-lead signals, we train the sparse matrix which represents a segment of ECG two-lead sample points to get thresholds of each individual. In these experiments, we define the threshold *ε*_*i*_ of the *i*th individual as

(8) $$\begin{array}{c} \varepsilon_{i}=R_{i}^{\min}-\delta , \end{array}$$

where *R*_*i*_^min⁡^ represents the minimum correlation coefficient in training set of the *i*th individual. *δ* is a variable that will be determined in the testing stage. To achieve the optimal identification result, we use increasing circulation *δ* to test result.

## 4. Experimental Results

We conducted a comprehensive experiment on public ECG databases, and we selected the MIT-BIH normal sinus rhythm database [25]. This database includes 18 long-term ECG recordings of subjects referred to the Arrhythmia Laboratory at Boston's Beth Israel Hospital. The subjects included in this database were found to have had no significant arrhythmias. These ECG data have a sampling rate of 128 Hz and a 12-bit binary representation.

For each individual, 8 segments of 10 sample periods long are obtained from the record of the ECG signal in the database. Thus, 1280 sample points in each segment are selected for frequency and rank order statistics. We set the matrix with 1300 rows and 1300 columns for reducing dimension easily. As we know, some of the ECG sample points are negative number, so every sample point value add 500 to get non-negative number. This modification of data set can avoid the problem for mapping them into the matrix. And then mapping those sample points into a 1300 × 1300 matrix. Next, we reduce the dimension of the matrix and store it as a sparse matrix.

In training stage, for each individual, there are 18 data sets for training via calculating the correlation coefficients. The process of training the neural network is from the MIT-BIH database for our sparse matrix experiment.

After training the ECG two-lead signals data with spare matrix correlation coefficient of 18 individuals, we can obtain the threshold of each individual. The test data for identification are also acquired from the same MIT-BIH database. For each individual, we recapture 10 segments of 1280 sample points of each lead in length. Note that these 10 segments are obtained at different locations of the ECG signal; that is, none of them overlap with previously selected segments used in the training process. Each segment is passed through the sparse matrix correlation coefficient classifier for the identification matching testing. Thus, there are 10 matching tests for each individual.

### 4.1. Measurement Approaches

We used two approaches to evaluate the algorithms.

#### 4.1.1. Success Rate

This is a metric used for accuracy measurement. Based on the results of comparisons between the individuals, when the correlation coefficient is smaller than the threshold correlation coefficient, we considered it as an identification error. Summing up these errors gives us the total number of errors; then we divided this figure by the total number of comparisons to give the success rate.

#### 4.1.2. False Acceptance (FA) and False Rejection (FR) Rates

These are also the metrics used for accuracy performance. The FR denotes the relative ratio of subjects which should be accepted but are actually rejected by the classifier; similarly, the FA is the ratio of subjects which should be rejected but are actually accepted by the classifier. The threshold which for FA/FR is obtained from the training set, was aimed to minimize.

### 4.2. Success Rate Results

Then, we use these to compute the correlation coefficient between testing data and template matrix of each individual to classify and identify the ECG testing data. As the *δ* of threshold defined is initialized by some random value, the performance might not be good enough; therefore, the classification should be trained a large number of times; for example, it is five times in our experiment. *δ* is initialized to zero, and then it increases by 0.01 to test the identification results. When *δ* increases to 0.20, we can find out the most appropriate *δ* for classification.

We use maximum correlation coefficient as prior method to calculate success rate. For a sparse matrix *ℳ* which came from one unidentified individual, we compute the correlation coefficient of this sparse matrix *ℳ* and each individual to get corresponding *R*_*i*_. Since there are 18 individuals in this experiment, we find out the max *R*_*i*_,  *i* ∈ (1,18). And then if *R*_*i*_ > *T*_*i*_, where *T*_*i*_ is the *i*th individual threshold that can refuse data not belonging to its own, we have *ℳ* which belongs to the *i*th individual. Else, *ℳ* does not belong to any individuals of those 18 individuals.

**Figure 3:**
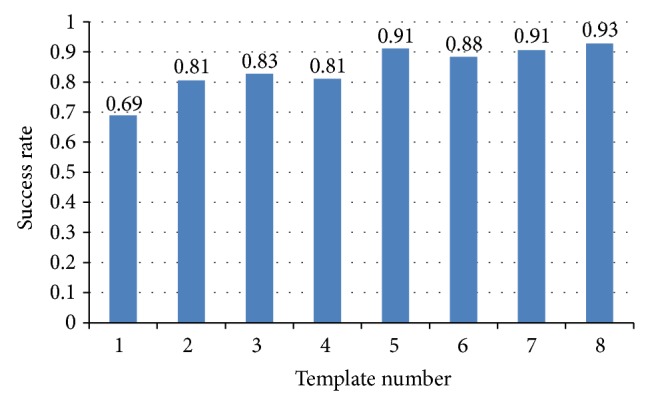
Maximum *R* prior success rate.

As we have 8 segments data for training, every segment can be a sparse matrix template for comparison during testing at the testing stage.  Figure 3 shows the success rate by using maximum correlation coefficient prior method to identify human with each sparse matrix template.

**Figure 4:**
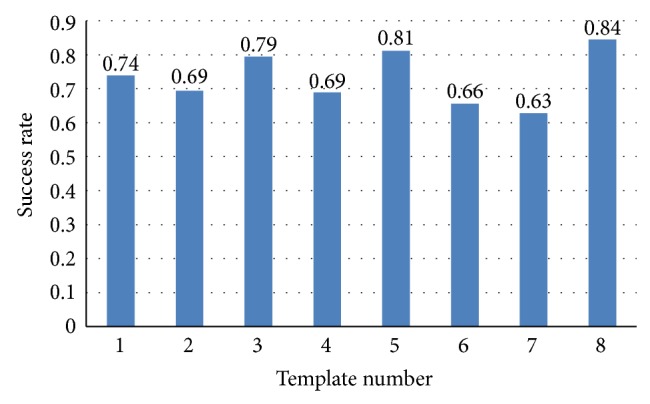
Least squares identification result.

Least square is another method for identifying human. For ECG signals which are extracted from MIT-BIH normal sinus rhythm database, we can get the correlation coefficient *R*_*i*_ corresponding to *i*th individual. After that, we compute the square Δ_*i*_:

(9) $$\begin{array}{c} {\Delta_{i}=\left(R_{i}-\epsilon_{i}\right)}^{2}, \end{array}$$

where *ϵ*_*i*_ means the average correlation coefficient of *i*th individual at training sets. And then we find out the minimum Δ_min⁡_ = min⁡⁡{Δ_*i*_∣*i* ∈ (1,18)}, where the Δ_min⁡_ corresponding individual is *j*. That is, Δ_min⁡_ = Δ_*j*_,  *j* ∈ (1,18). As a result, this ECG signal belongs to *j*th individual.  Figure 4 shows the success rate by using least squares correlation coefficient prior method to identify human with each template sparse matrix.

#### 4.2.1. FA and FR Rates Results

**Figure 5:**
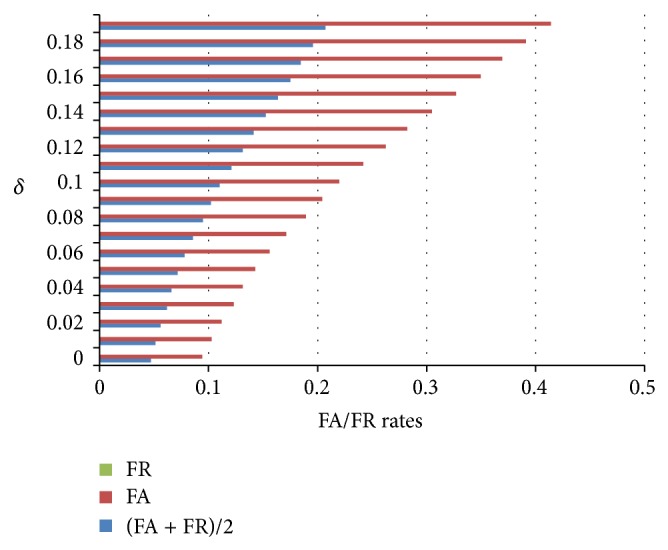
FA and FR rates.

Figure 5 summarizes the FA and FR change with the *δ* changes. It shows that the FA/FR ratio for the matching test lies between 0.047 and 0.207 for 18 individuals with 10 segments each, which is acceptable for multiple subject classification.

The FA/FR rate of Figure 5 is compared with the 7th template of each individual. Next, we will show experiment result of different templates.

**Figure 6:**
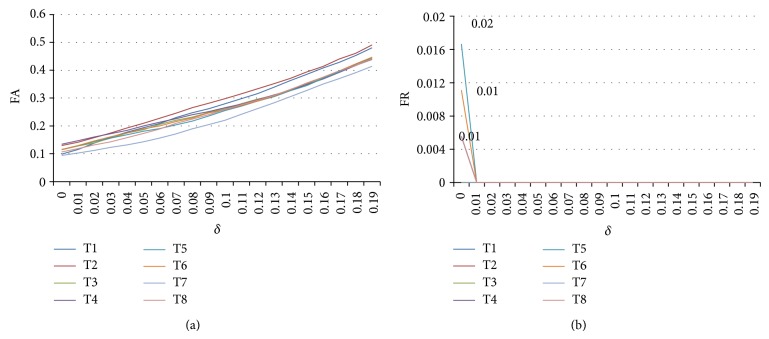
(a) FA rates of eight templates with different threshold and (b) FR rates of eight templates with different threshold.

Figure 6(a) displays different FA rates of those eight templates of the sparse matrices. And we can figure out that the FA rate is smaller for all templates when the threshold *δ* is small. The best is *δ* = 0.

Figure 6(b) displays different FR rates of those eight templates of sparse matrices. And we can figure out that the FR rate is smaller for all templates when the threshold *δ* is bigger. We have that when *δ* ≥ 0.01, all templates FR rate is zero.

The Acc accuracy of sparse matrix correlation coefficient algorithm can be calculated by the FA rate and FR rate. In common, the calculation function would be

(10) $$\begin{array}{c} \text{Acc}=1-\frac{\text{FA}+\text{FR}}{2}. \end{array}$$

Although FA rate and FR rate have different tendency when changing threshold *δ*, we might find out a suitable *δ* by an iterative loop to find a better accuracy Acc.

According to formula (10), we can get higher Acc when (FA + FR)/2 is smaller.

**Figure 7:**
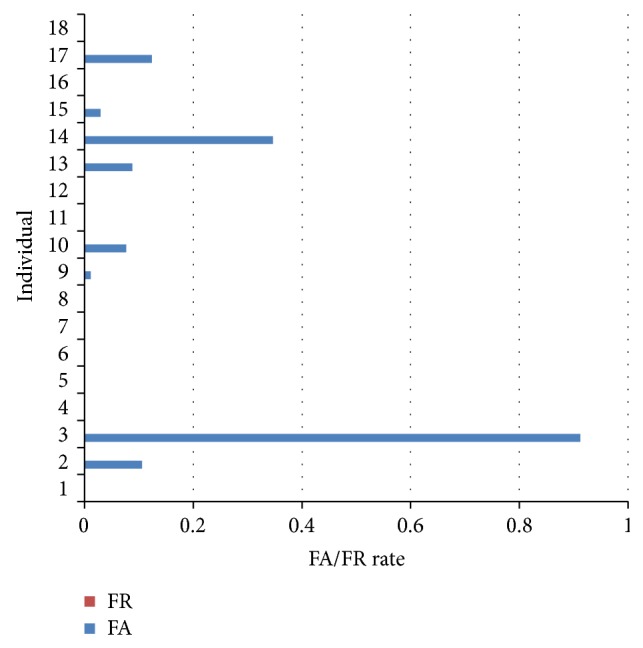
FA and FR of sparse matrix with correlation coefficient computation.

As shown in Figure 7, we have eight templates matrix for training and testing ECG data in our SMCC algorithm. When *δ* = 0, the result can reach the better performance. So we choose templates *T* = 7 and *δ* = 0 to show FA and FR of each individual by sparse matrix correlation coefficient algorithm here, as Figure 7 shows.

## 5. Comparison

To compare with the common one-dimensional algorithm, three ECG identification algorithms are compared in this experiment with the same database and comparison method. The four common identification algorithms are described below.

First, we list the ECG identification algorithms with single lead signal data.

### 5.1. Comparing with Reduced Binary Pattern (RBP) Algorithm

This algorithm uses the frequency and rank order statistics of the input ECG signal [26]. For any ECG signal, we can express it as {*x*_1_, *x*_2_, *x*_3_,…, *x*_*n*_}, where *x*_*i*_ represents the *i*th signal from the input data. According to the decrease or increase of two consecutive *x* values, the two-state function, *R*_*n*_, is mapped onto the values of 0 and 1, respectively:

(11) $$\begin{array}{c} R=\left\{\begin{array}{cc} 0, & x_{n}\leq x_{n-1},\\ 1, & x_{n}\leq x_{n-1}. \end{array}\right. \end{array}$$

Through formula (11), the reduced binary pattern is simply represented by one binary sequence consisting of digits 0 and 1.

In *w*_*k*_2^*m*^ − 1*w*_*k*_ *S*_1_ *S*_2_ counting and ranking process, the frequency of each *w*_*k*_ whose value ranges between 0 and 2^*m*^ − 1 is calculated in the counting process. Therefore, we incorporate a weighted distance formula to define the measurement of similarity between *S*_1_ and *S*_2_:

(12) $$\begin{array}{c} D_{m}\left(S_{1},S_{2}\right)=\frac{\sum_{k=0}^{2^{m}-1}\left|R_{1}\left(w_{k}\right)-R_{2}\left(w_{k}\right)\right|p_{1}\left(w_{k}\right)p_{2}\left(w_{k}\right)}{\left(2^{m}-1\right)\sum_{k=0}^{2^{m}-1}p_{1}\left(w_{k}\right)p_{2}\left(w_{k}\right)}, \end{array}$$

where *p*_*i*_(*w*_*k*_) and *R*_*i*_(*w*_*k*_) represent the probability and ranking of *w*_*k*_ in the sequence *S*_*i*_,  *i* = 1  or  2. The absolute difference between two rankings is multiplied by the normalized probabilities as a weighted sum; the factor 2^*m*^ − 1 in the denominator is to ensure all values of *D*_*m*_ lie within the scope of [0, 1].

### 5.2. Comparing with Waveform Algorithm

In a waveform-based study [8], a total of 19 features are extracted from the four classes: amplitude (PQ, RQ, TQ, RT, PS, RP, TS, RS, PT, and QS), duration (QS, PR, QR, ST, and QT), slope (RS, ST, and QR), and area (area of the QRS triangle). These features form a feature vector *S*.

After obtaining some waveform feature for the individual difference, we use formula (12), a similarity algorithm to evaluate difference between two individuals. The closeness between two feature-vectors *S*_1_ and *S*_2_ is considered as their distance *d*(*S*_1_, *S*_2_); the intra- and intergroup distances can be evaluated through (12).

### 5.3. Comparing with Wavelet Transform Algorithm

Wavelet analysis or wavelet transformation is the finite or rapid attenuation of oscillation waveform signals, which is called the mother wavelet.

The procedures of the wavelet-based algorithm [9] in comparison include the following: each R-R cardiac cycle is obtained through R-R detection; an interpolation is performed on the R-R interval so each R-R cardiac cycle holds 284 data points; every R-R cycle is cut into three parts, each containing 85, 156, and 43 points; the first 85 and the last 43 points in each R-R cycle are assembled to form a 128-point segment; every four segments are grouped and an *n*-level discrete wavelet transform (DWT) is performed to obtain the corresponding wavelet coefficients. Four of the computed wavelet coefficients are gathered as a wavelet vector and expressed as

(13) $$\begin{array}{c} S=\left[a_{n},d_{n},d_{n-1},d_{1}\right]. \end{array}$$

The Euclidean distance between two wavelet vectors *S*_1_ and *S*_2_ is regarded as their distance  *d*(*S*_1_, *S*_2_); the intra- and intergroup distances can then be calculated through (12).

So far, we have introduced three algorithms for human identification by using one lead of ECG signal. Now we will design an experiment to conduct a comparison between the three algorithms and our sparse matrix algorithm.

In the evaluation using the MIT-BIH normal databases, it is obvious from the comparison of outcomes shown in Table 1 that the RBP, waveform-based, and wavelet transform algorithms perform well but our advanced sparse matrix with two-lead algorithm still excels them, and it has a better accuracy rate in the MIT-BIH normal public database.

### 5.4. Comparison Result with One-Lead Methods

From Table 1 we know that using two-lead ECG signal for human identification can enhance the identification accuracy. The result demonstrates that there is a great potential of our proposed method in the ECG biometrics system. Next, we will compare two typical two-dimension algorithms with our two-lead ECG algorithm.

### 5.5. Comparing with Basic Two-Dimensional Method

In Section 3, we described the basic flow of our sparse matrix algorithm with two-lead ECG signal. We reduce the sample points dimension directly through formula (4). Now we propose the basic method to deal with the similarity of two sparse matrices.

As we know, the baseline to measure similarity between two matrices is subtracted for two sparse matrices SM_1_ and SM_2_, let

(14) $$\begin{array}{c} b_{m}={\left({\text{SM}}_{1}-{\text{SM}}_{2}\right)}^{2}, \end{array}$$

and then calculate the correlation of *b*_*i*_ and *b*_*j*_. The following steps to calculate the FA and FR are the same as our sparse matrix.

### 5.6. Comparing with PCA Method

In this comparison, we use principal component analysis (PCA) to fusion two-lead ECG signal for identification. PCA is a statistical technique whose purpose is to condense the information of a large set of correlated variables into a few variables as principal components, while not throwing overboard the variability present in the data set [27].

For a matrix *X* which is consisting of sample data. The linear transformation of converting *X* to *Y* is

(15) $$\begin{array}{c} Y=PX, \end{array}$$

where *Y* is considered as extracting the principal components of the original matrix *X*. And *P* is a linear transformation matrix. Each row of *P* is the eigenvector of matrix *C*_*X*_, and

(16) $$\begin{array}{c} C_{X}=\frac{1}{n}XX^{T}. \end{array}$$

As our method, in this PCA algorithm, we read two-lead signal with the same steps. The difference is that PCA algorithm adds the second lead data behind the first lead and then use PCA method to reduce the features. Choose the main feature to train and test for human identification.

### 5.7. Comparison Result with Two-Lead Methods

From our experiment, we determined that when we choose the main 19 features, the result of identification is improved over existing approaches.

**Figure 8:**
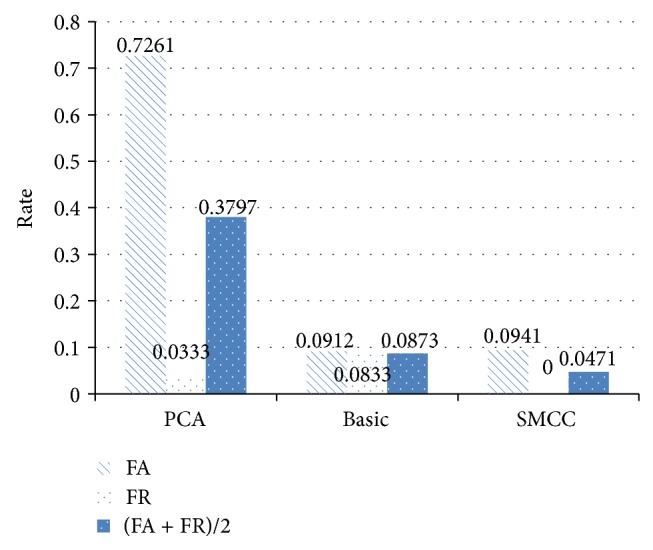
Comparison of ECG two-lead algorithms.

Figure 8 shows the comparison of those two algorithms and our sparse matrix algorithm.

We can get our SMCC algorithm which have the better FA/FR rate compared with other two two-lead ECG identification methods.

## 6. Conclusions

In this paper, a new ECG identification method is proposed with two-dimensional sparse matrix algorithm, in which two-lead ECG signals are fused using a sparse matrix approach. Using experimentation, we demonstrate that two-lead identification offers improvements over one-lead identification. Two typical two-dimensional classifications were compared with our method. The performance of our sparse matrix has around 95.3% accuracy which is better than basic two-dimensions 90.1% and PCA 63.2%. The results show that our sparse matrix has a good performance for ECG identification.

## Figures and Tables

**Table 1:** FA/FR of the compared result.

Item	RBP	Waveform	Wavelet	SMCC
FA	0.3748	0.3092	0.3734	0.0941
FR	0.2500	0.1222	0.0833	0
(FA + FR)/2	0.3124	0.2157	0.2283	0.0471
Accuracy	68.76%	78.43%	77.17%	95.29%
